# Hysterical Mania

**Published:** 1883-03

**Authors:** W. B. Kesteven


### HYSTERICAL MANIA: ATHEROMATOUS DISEASE OF LEFT INTERNAL CAROTID: THROMBOSIS OF ARTERIES AT BASE OF BRAIN, WITH CONSEQUENT SOFTENING OF CEREBRAL SUBSTANCE. By W. B. KESTEVEN, M.D.

The following case presents points of interest, both psychologically and pathologically:

On the 30th of September last, I was requested to undertake the care of a lady who was said to he suffering from a severe form of hysteria. The patient came to me in the afternoon of that day, was perfectly collected and cheerful, entered into conversation in the intelligent and self-possessed manner of an educated lady. She joined our family party at tea, and in the course of the evening sat down beside a whist table, to watch the game, as she herself said. In about half an hour she suddenly left the room and retired to her bedroom, where, on being followed, within a very short time, she was found almost in a state of nudity, complaining that her bowels would not act, and endeavoring to evacuate the rectum with her fingers. She talked incoherently, was incessantly moving about; so restless that it was impossible to keep her in bed by any persuasion, although at the same time declaring herself paralyzed; she passed her evacuations under her. On the following morning she became quieter, kept to her bed the greater part of the day, but was still incoherent. She took no food except by strong persuasion. On the morning of the third day, while standing up to be dressed she suddenly fell to the ground, but got up again, all the while protesting that she had lost the use of her legs. She seated herself on the floor in the corner of the room, so as to support herself on either hand by the angles of the wall. She was cheerful, even loquacious, for a few hours.

It became necessary to remove her to an asylum, which was effected by carrying her, as it appeared that she would not, or could not, stand. The sequel shows that she could not. As the case, however, had previously been regarded as one of hysteria, her want of muscular power was doubted. Dr. Wright, of Northumberland House, Finsbury Park, under whose care she was placed on Oct. 2nd, has obliged me by notes of her condition whilst under his observation. On admission, she was semicomatose, and in a state of profuse perspiration. She did not move or speak. She would half-open her eyelids and gaze furtively around; her eyes were turned to the left. She seemed to be, to a great extent, aware of what was going on. She gave the impression that she was simulating paralysis and stertorous breathing. Her right side was "limp," but no marked difference from the state of the left side. No reflex action elicited on soles of the feet. Before she died there was a distinctly paralytic state of the left cheek and ptosis of the left eye. Her temperature rose to 103°, and her pulse to 130 during the day of her death, Oct. 4th. Dr. Wright has kindly furnished me with a copy of Dr. Goodhart's report of the macroscopic examination of the brain.

INSPECTION MADE 4 P. M., OCTOBER 5, 1882.

*Cranial Bones.*—Thin.

*Dura Mater and Sinuses.*—Healthy, except that dura mater was unduly adherent to skull.

*Arachnoid and Pia Mater.*—All looked quite healthy, but when stripped from the gray matter beneath on the left side the surface beneath was left ragged and soft; the color being unduly yellow, from the intermixture of blood pigment.

The *vessels* were all healthy except one, and that the left internal carotid. This vessel was obviously plugged by firm substance, and on making a transverse section of this part and examining it closely, it became evident that the coats of the vessel were extensively diseased here: that atheromatous thickening had occurred, leading to considerable diminution of the caliber of the vessel. This had, of course, been going on for some time, probably for many months. More recently, perhaps in the last two or three weeks, perhaps even more lately than this, the diminished channel had become completely closed by fresh clots being deposited upon the roughened arterial wall.

The transverse section of the artery thus showed an outer thick yellow zone, or partial zone, and an inner claret-colored part.

Looked at from above, the left hemisphere was makedly fuller than the right, and bulged out, more particularly in the lower part of the anterior, central, or ascending frontal convolution. It was in this part that the membranes, when stripped, left a ragged surface behind. Making sections of this hemisphere, it was found that the central cortex about this convolution—that is to say, it and the convolutions adjacent—was extensively swollen, so as to have lost its outline of demarcation from the white matter beneath ; it was echymosed in many places, minutely vascular in many more, and everywhere had lost its consistence when compared with the opposite hemisphere and the sounder parts of this one.

The lenticular nucleus, the corpus striatum, had suffered in the same way—bloated-looking and pulpy.

It was particularly noticed, and this has an important bearing on the symptoms, that except in the superficial layers of the gray matter, which were beginning to disintegrate, there was as yet no evident solution of continuity of the brain-fibers, and thus there had been no marked paralysis.

The ventricles and other parts of brain all looked healthy.

To the preceding I would add the results of my microscopical examination of portions of the convolutions taken from near the seat of the softened substance. These may be summed up as disintegration of structure by softening—atrophy of nerve-cells, dilatation of the minute vessels, and extensive miliary degeneration—conditions, all of which are indicative of longer-standing disease than would appear from the history of the case. The patient had always been regarded as hysterical. In the month of June last she had a transient attack of hemiplegia. After this she experienced paroxysms of hysteria, bordering upon mania ; she complained of tingling sensations in various parts of her body.

This case is worthy of note on account of the difficulty of diagnosis in its early stages, and the contrast presented between its later symptoms, compared with the extent of pathological lesions revealed after death.—*Brain.*

